# Examining the Role of Asef and Epidermal Growth Factor on Cell Migration and Proliferation in Triple Negative Breast Cancer Epithelial Cells

**DOI:** 10.17912/micropub.biology.002036

**Published:** 2026-06-24

**Authors:** Natalie Alaniz, Khameeka N. Kitt

**Affiliations:** 1 Biology Department, Saint Mary's College of California, Moraga, CA, United States

## Abstract

Cell proliferation and migration are cellular processes essential to normal cell function; errors in these cellular processes impact the ability of a cell to maintain cellular homeostasis. The current study examines the role of Asef, a GEF for Rac1, in cell proliferation and migration in the presence or absence of EGF in a triple negative breast cancer epithelial cell line. Overexpression of Asef reduced stress fiber formation and increased cell migration and proliferation in MDA-MB-231 epithelial cells. These data indicate a role for Asef in the EGFR/Rac1 signaling cascade involved in regulating cellular behavior.

**Figure 1. Asef-HA overexpression in MDA-MB-231 breast epithelial cancer cells causes changes in protein localization, cell migration, and cell proliferation:** Human MDA-MB-231 breast cancer epithelial cells were transfected with Asef-HA (green) and allowed to express for 24 hours before treatment with 10 ng/ml of EGF for 24 hours. (A) In the absence of EGF, Asef and Rac1 (red) localize to the cytoplasm and membrane. In the presence of EGF for 24 hours, Rac1 colocalizes with Asef at the cell membrane (arrows). Scale bar: 40 µm. (B) Asef localizes to the cytoplasm and membrane ruffles (arrows) in cells with and without EGF treatment. In the absence of EGF, EGFR (red) localizes to the cell membrane and cytoplasm in Asef-HA expressing cells (arrowhead). Cells treated with EGF for 24 hours caused a minor shift in EGFR localization from the membrane to vesicle-like structures inside the cell (arrowhead). Scale bar: 40 µm. (C) Asef localizes to the cytoplasm and membrane ruffles (arrows) in cells with and without EGF treatment. Cortical Actin (red) appears unchanged in Asef-HA expressing cells (arrowhead). Cells expressing Asef-HA show reduced stress fibers extending across the cell (asterisks) compared to untransfected cells (arrows) in the presence or absence of EGF for 24 hours. Scale bar: 40 µm. (D) MDA-MB-231 cell monolayers overexpressing HA-only or Asef-HA were scratched and imaged over 24 hours in the presence or absence of EGF. The width of the wound was measured using ImageJ and plotted to examine wound width over time. Asef-HA expressing cells, regardless of EGF treatment, migrated faster than control cells; however, Asef-HA expressing cells without EGF treatment showed a significant increase in migration at 12 and 24 hour time points compared to HA only expressing cells. (**P < 0.005; n=3 measurements of each group). (E) MDA-MB-231 cell monolayers overexpressing HA-only or Asef-HA were serum-starved and treated with 10 ng/ml of EGF for 24 hours. Following EGF-treatment, MTT reagent was added, cells were solubilized with DMSO, and absorbance was read at 570 nm. Asef-HA expressing cells in the absence of EGF treatment showed a significant increase in cell proliferation compared to untransfected cells (*P < 0.05; n=3 measurements of each group). (F) Model of the Rho GTPase signaling pathway demonstrating how Asef could be recruited downstream of EGFR signaling to regulate Rac1 activation and stimulate cell proliferation and migration.

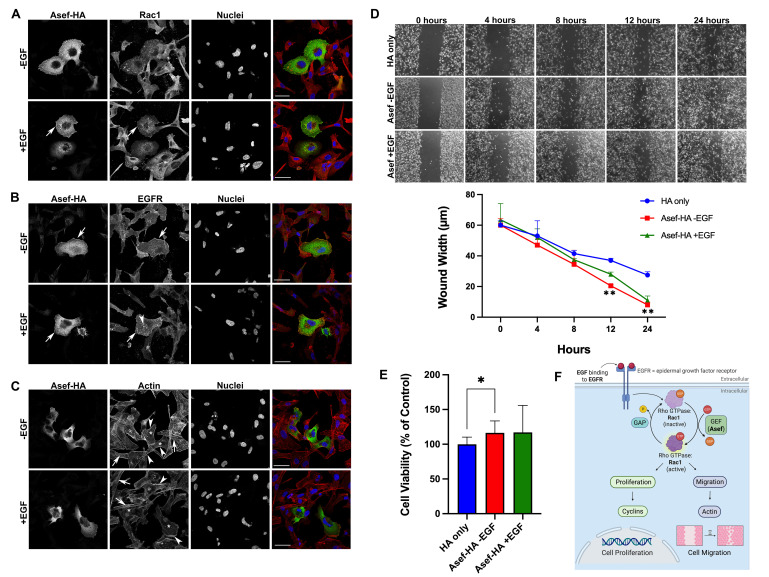



## Description

Breast cancer is not a single disease, but rather a group of diverse diseases with different subtypes. Breast cancers are organized into subtypes distinguished by the absence or presence of certain proteins on the cancer cells (Fragomeni et al., 2018). Triple negative breast cancers (TNBCs) are a subtype of breast cancer characterized by the absence of three receptors: estrogen receptor (ER), progesterone receptors (PR), and human epidermal growth factor 2 (HER2) (Yin et al., 2020). The TNBC subtype is both invasive and aggressive and occurs more frequently in younger women and African American women (Hines et al., 2011). Understanding the specific subtype of breast cancer is crucial for determining the most effective treatment options. The ductal carcinoma TNBC epithelial cell line, MDA-MD-231, is commonly used to study invasive breast cancer and metastasis due to its highly invasive and migratory nature (Amaro et al., 2016). 

Cell adhesion, proliferation and migration are three critical cellular processes required for tissues to maintain homeostasis. When one or all of these cellular processes become unregulated, for example in cancer, this can lead to uncontrolled cell growth and metastasis (Hanahan & Weinberg, 2011). Various molecules have been identified that are involved in maintaining the balance of these cellular processes under normal conditions. Rho GTPases are a family of proteins that act as molecular switches that can be activated by guanine nucleotide exchange factors (GEFs) (Lawson & Ridley, 2017). The Rho GTPase Rac1 has been shown to regulate the actin cytoskeleton and cell proliferation by working downstream of the receptor tyrosine kinase, epidermal growth factor receptor (EGFR) and its ligand, epidermal growth factor (EGF), to regulate various kinase pathways (Dise et al., 2008; Itoh et al., 2008). Dimerization of EGFR leads to the phosphorylation of different proteins along these pathways involved in regulating gene expression and activation of proteins involved in promoting cell migration, adhesion, and proliferation (Wee & Wang, 2017). Adenomatous Polyposis Coli (APC)-stimulated guanine nucleotide-exchange factor, Asef, is a GEF that activates Rac1 to promote cell migration by reducing cell-cell adhesions and remodeling the actin cytoskeleton (Kawasaki et al., 2000, 2010). In colorectal cancer, APC can activate Asef, leading to enhanced migration and invasion (Mitin et al., 2007). 

The MDA-MB-231 cell line serves as a valuable model for studying breast cancer proliferation and invasion due to the presence or absence of molecules involved in maintaining normal cell function. MDA-MB-231 cells express Rac1 and are responsive to EGF stimulation (Davidson et al., 1987; Veber et al., 1994). However, it is currently unknown how EGFR activation impacts the regulatory molecules, Asef and Rac1, along with cell migration and proliferation in MDA-MB-231 cells. The current study aims to examine the role of Asef in cell proliferation and migration in the presence of EGF in MDA-MB-231 cells. We hypothesize that due to the regulatory nature of Asef on Rac1, overexpression of Asef in the presence of EGFR signaling pathway will lead to changes in the protein localization and an increase in proliferation and cell migration due to activation of downstream signaling pathways. 

Asef-HA (Kawasaki et al., 2000) was overexpressed in MDA-MB-231 cells using a lipid-based transfection approach and protein localization of Rac1, EGFR, and actin was observed in untreated and EGF-treated cells. Using immunofluorescence, Asef-HA overexpressing cells show Asef both in the cytosol and at the cell membrane co-localizing with endogenous EGFR and Rac1 protein in untreated cells and cells treated with EGF for 24 hours ( Figure 1A- B). The increased protein localization for Asef and Rac1 at the membrane suggests a possible interaction of the proteins to control cell migration ( Figure 1A ). Kawasaki, et al. previously showed that Asef promotes the activation of the Rac1 protein, exchanging GDP for GTP, in endothelial cells (Kawasaki et al., 2010). Additional studies are required to determine if there is a direct interaction between the overexpressed Asef-HA protein and Rac1-GTP at the membrane.

EGFR protein appears to internalize into small vesicular structures after EGF stimulation ( Figure 1B ), suggesting activation of the receptor and downstream cellular pathways (Pennock & Wang, 2003). Due to the shift of EGFR from the membrane to internal vesicular structures in the presence of EGF and previous studies which have examined the impact of EGFR internalization on actin remodeling and cell migration (Pinilla-Macua et al., 2025), actin organization in Asef-HA expressing cells was examined. Actin organization provides a critical scaffold to cellular organization and structure and is represented by two different and interconnected cytoskeletal structures: cortical and stress fibers (Vallenius, 2013). Cortical actin forms a meshwork for actin filaments beneath the cell membrane and is involved in controlling cell shape by locally modifying cortical tension (Chalut & Paluch, 2016). Stress fibers are organized as bundles of actin filaments and play a role in cell migration, adhesion, and mechanosensing, particularly in the context of cancer. These structures are involved in cell migration and linked to cell stiffness, which can either promote or inhibit migration depending on the specific context and cell type (Fischer et al., 2021; Tavares et al., 2017). To determine the impact of Asef on actin filament organization, MDA-MB-231 cells were transfected with Asef-HA. Stress fibers appear to be reduced in Asef-HA expressing cells (asterisks), with or without EGF, as compared to cortical actin, which remains unchanged in both untreated and EGF-treated cells ( Figure 1C ). The loss of stress fiber formation in Asef-HA cells in comparison to untransfected cells suggests the cells have lost their stability and may be more susceptible to movement.

The lack of stress fiber formation in Asef-HA expressing cells suggests that the cells may be more migratory in nature. To determine if overexpressed Asef leads to an increase in cell migration, a wound healing assay was performed in MDA-MB-231 cells overexpressing Asef-HA. Data indicate that cells overexpressing Asef-HA with or without EGF for 24 hours migrate faster compared to HA only expressing cells ( Figure 1D ). Quantitative analysis shows a significant increase in proliferation at 12 and 24 hours between HA-only cells and Asef-HA without EGF stimulation in comparison to cells under EGF stimulation ( Figure 1D ). These data support the hypothesis that overexpression of Asef-HA in MDA-MB-231 cells activates pathways that lead to increased cell migration, by possibly impacting actin organization and turnover at the leading edges of cells (Tian et al., 2015). This is further validated by the reorganization and distribution of key molecules involved in migration, namely EGFR, Rac1, and actin filament organization ( Figure 1A- C) and that Rac1 serves as a relay protein between EGFR and cell migration (Dise et al., 2008). The lack of significance between HA-only cells and Asef-HA with EGF treatment suggests that the EGF concentration was not sufficient to induce a response. Previous studies have shown that EGF at a 10 fold higher concentration appears to have more of an impact on MDA-MB-231 cell migration (Kozlova et al., 2016) than lower concentrations. Future studies will determine if higher EGF concentrations lead to significant changes in cell proliferation and migration over basal conditions in cells overexpressing Asef.

Internalization of EGFR via EGF binding, as observed in Figure 1B, may lead to activation of cyclins and/or other cell division regulators to promote an increase in cell proliferation. Furthermore, the EGFR pathway is frequently implicated in cancer development and progression, as it can drive uncontrolled cell growth and division (X. Song et al., 2020; Z. Song et al., 2016). To determine if the activation of EGFR leads to possible increase in MDA-MB-231 cell proliferation due to downstream pathways becoming activated, a MTT assay was performed on control and Asef-HA transfected cells. Data show a significant increase in proliferation in Asef-HA without EGF-stimulation compared to HA-only cells and there was no significant change in Asef-HA with 10 ng/ml EGF for 24 hours ( Figure 1E ). Similar to the wound healing assay, the lack of significance between control cells and Asef-HA with EGF treatment suggests that the EGF concentration may not be sufficient. These data support the hypothesis that overexpression of Asef-HA in MDA-MB-231 cells may activate pathways that lead to cell proliferation (Lo & Hung, 2006; Pennock & Wang, 2003). Additional studies are required to determine which pathways are responsible for the increased cell proliferation and the impact of EGFR signaling on cell proliferation.

Our findings show that Asef overexpression impacts actin filament remodeling and increases cell migration and proliferation in a metastatic breast cancer cell line. Mutated versions of APC and Asef have been implicated in colon cancer cell migration and invasion (Yang et al., 2021), and we extend the role of Asef into a highly invasive and aggressive epithelial breast cancer subtype. Asef is primarily known for its role in APC-Wnt signaling (Kawasaki et al., 2000); however, Asef and Rac1 were shown to be involved in the EGFR pathway and may be a possible molecular target to consider in understanding the aggressiveness of triple negative breast cancer (Itoh et al., 2008). Future work aims to explore the connection of Asef to signaling proteins involved in controlling cell proliferation and how Asef regulates Rac1 and actin organization in the presence of EGF to control cell motility. 

## Methods

Cell Culture 

Triple negative ductal breast carcinoma epithelial cell line MDA-MB-231 (ATCC) were grown in Dulbecco's Modified Eagle Serum (DMEM) high glucose media (Thermo Fisher Scientific) with 10% fetal bovine serum and 1% penicillin-streptomycin-glutamine (Thermo Fisher Scientific). Cells were incubated under the following conditions: 5% CO _2_ and 37˚C.

**Transfection**

MDA-MB-321 cells were transfected with pcDNA 3.1-HA Asef (human) full length plasmid (provided by Tetsu Akiyama lab, University of Tokyo, (Kawasaki et al., 2000) ) using Lipofectamine 2000 diluted in Opti-MEM (Thermo Fisher Scientific) following product recommendations. As a control for proliferation and scratch assays, cells were transfected with pcDNA 3.1-HA plasmid alone. Transfected cells were incubated for 24 hours in antibiotic-free media before being replated in various well formats for various assays.

**Immunofluorescence**

Transfected MDA-MB-231 cells (with or without Asef-HA) were placed on rat tail collagen-coated coverslips in serum-free media (DMEM with 1% penicillin-streptomycin-glutamine) and treated with or without 10 ng/mL of EGF for 24 hours. Cells were fixed using 4% paraformaldehyde 48 hours post-transfection. To localize HA, Rac1, and EGFR, cells were blocked and labeled with primary antibodies for HA (Roche), Rac1 (BD Biosciences) and EGFR (Thermo Fisher Scientific) followed by secondary antibodies conjugated to Alexa Fluor 488 and 594 (Thermo Fisher Scientific). Actin filaments and nuclei were labeled using Phalloidin 594 and Hoechst 33342 DNA stains, respectively (Thermo Fisher Scientific). Cells were imaged using the Leica STELLARIS Inverted Spectral Live Cell Confocal Microscope. 

**Scratch Assay (Wound Healing)**

Transfected MDA-MB-231 cells (with or without Asef-HA) were placed on rat tail collagen-coated 12 well plates and allowed to form a monolayer in serum free media. Monolayers were scratched 24 hours later using a sterile 200 µL pipette tip, washed using phosphate-buffered saline (PBS) to remove debris, and incubated in serum-free media treated with or without 10 ng/mL of EGF for 24 hours. Images were captured at 0, 4, 8, 12 and 24 hours. The area between both sides of the scratch were quantified after each image time stamp to determine the wound closure distance using ImageJ software. Distance of wound closure for each group at each time point was measured in µm using ImageJ (pixels per micron was determined using scale bar) and plotted to display the width of the wound closure over time. 

**Proliferation (MTT) Assay**

Transfected MDA-MB-231 cells (with or without Asef-HA) were placed in a 96-well tissue culture-treated plate in serum free media. Cells were treated with or without 10 ng/mL of EGF for 24 hours before adding 10 µL of a 5 mg/mL stock of MTT reagent per well and incubated for three hours. After three hours, the MTT reagent was removed and 100µL DMSO was added to each well to solubilize cells, and the optical density was measured at 570 nm (Tecan Plate Reader). Percent viability was calculated for each well and normalized against the control (HA-only). 

**Statistical Analysis**

For the scratch assay, a multiple unpaired t-test analysis was performed to determine the significance at each time point. Three biological replicates (independent wells) were analyzed for each group. For the proliferation (MTT) assay, an unpaired t-test was used to determine significance between the treated groups compared to the control. Three biological replicates (independent wells) were analyzed for each group. All data was graphed using GraphPad Prism. 

## Reagents

**Table:** 

**Plasmid **	**Description **	**Source**
pcDNA 3.1-HA- Asef	Full length Asef (human)	Tetsu Akiyama lab, University of Tokyo
**Cell Line **	**Description **	**Source**
MDA-MB-231	Human Epithelial Breast Tissue isolated from Mammary Gland; Adenocarcinoma	ATCC Catalog #: HTB-26
**Antibodies **	**Dilution **	**Source**
Phalloidin 594	1:400	Thermo Fisher Scientific Catalog #: A12381
Alexa Goat anti Rabbit 488	1:800	Thermo Fisher Scientific Catalog #: A-11008
Alexa Goat anti Mouse 594	1:800	Thermo Fisher Scientific Catalog #: A-11005
HA (Rabbit)	1:500	Abcam Catalog #: ab9110
Rac1 (Mouse)	1:200	BD Biosciences Catalog #: 610651
EGFR (Mouse)	1:200	Thermo Fisher Scientific Catalog #: MA513269
Hoechst 33342 DNA stain	1 µg/ml final	Thermo Fisher Scientific Catalog #: H3570
